# Supplementary material for: Folate Status as a Nutritional Indicator among People with Substance Use Disorder; A Prospective Cohort Study in Norway
Source: Int J Environ Res Public Health. 2022 May 9;19(9):5754. doi: 10.3390/ijerph19095754 (PMC9099634; doi:10.3390/ijerph19095754)
Supplement: Supplementary file 1 [file ijerph-19-05754-s001.zip › Supplementary.pdf]

Supplementary Table S1 – Scoring scheme of the "substance use severity score".

	**Frequency of Use**					
**Substance**	Never	< 1x/month	1–3x / month	1–3x/ week	4–6x/ week	Daily
Alcohol	0	1	2	3	4	5
Cannabis	0	1	2	3	4	5
Stimulants	0	1	2	3	4	5
Non-prescribed opioids	0	1	2	3	4	5
Benzodiazepines	0	1	2	3	4	5

Substance use severity score = sum of scores on each substance (min 0, max 25).
